# *Agrobacterium*-Mediated *Capsicum annuum* Gene Editing in Two Cultivars, Hot Pepper CM334 and Bell Pepper Dempsey

**DOI:** 10.3390/ijms22083921

**Published:** 2021-04-10

**Authors:** Sung-il Park, Hyun-Bin Kim, Hyun-Ji Jeon, Hyeran Kim

**Affiliations:** 1 Interdisciplinary Graduate Program in BIT Medical Convergence, Kangwon National University, Chuncheon 24341, Korea; stonebridge0@kangwon.ac.kr; 2 Department of Biological Sciences, Kangwon National University, Chuncheon 24341, Korea; 201412602@kangwon.ac.kr (H.-B.K.); msy2b@kangwon.ac.kr (H.-J.J.)

**Keywords:** CRISPR/Cas9, pBAtC binary vector, *CaMLO2*, *Capsicum annuum* CM334, *Capsicum annuum* Dempsey, *Agrobacterium tumefaciens*

## Abstract

Peppers (*Capsicum annuum* L.) are the most widespread and cultivated species of Solanaceae in subtropical and temperate countries. These vegetables are economically attractive worldwide. Although whole-genome sequences of peppers and genome-editing tools are currently available, the precision editing of peppers is still in its infancy because of the lack of a stable pepper transformation method. Here, we employed three *Agrobacterium tumefaciens* strains—AGL1, EHA101, and GV3101—to investigate which *Agrobacterium* strain could be used for pepper transformation. Hot pepper CM334 and bell pepper Dempsey were chosen in this study. *Agrobacterium tumefaciens* GV3101 induced the highest number of calli in cv. Dempsey. All three strains generated similar numbers of calli for cv. CM334. We optimized a suitable concentration of phosphinothricin (PPT) to select a CRISPR/Cas9 binary vector (pBAtC) for both pepper types. Finally, we screened transformed calli for PPT resistance (1 and 5 mg/L PPT for cv. CM334 and Dempsey, respectively). These selected calli showed different indel frequencies from the non-transformed calli. However, the primary indel pattern was consistent with a 1-bp deletion at the target locus of the *C. annuum* *MLO* gene (*CaMLO2*). These results demonstrate the different sensitivity between cv. CM334 and Dempsey to *A. tumefaciens*-mediated callus induction, and a differential selection pressure of PPT via pBAtC binary vector.

## 1. Introduction

Owing to global climate change and the increase in participation of the older adult population in agriculture, facility agriculture is gaining importance. Its practice has increased worldwide. To improve sustainability, horticulture facilities are shifting to conventional plant–microbe interactions, resulting in several newly emerging plant diseases, such as powdery mildew infection in tomato and pepper [1]. Symptoms of the infection can be macroscopically observed as white-covered epithelial mycelia of powdery mildew pathogens on leaves and fruits [2]. Some biotrophic plant pathogens, including powdery mildew fungi, display properties that pose challenges to conventional infection prevention methods, such as protectant fungicides, so resistant cultivars are needed. Powdery mildew resistance conferred by mildew resistance locus O (*MLO*) genes has been reported in various plant species, such as barley, *Arabidopsis*, and wheat [3,4,5].

Whole-genome sequences of peppers have been available since 2014 [6,7]. We have discovered that peppers have extended resistance genes compared to other species in Solanaceae [8,9]. Recently, we reported CRISPR/RNPs-based precise *Capsicum annuum MLO* gene (*CaMLO2*) editing in both callus-derived protoplasts and leaf protoplasts from hot pepper and bell peppers [10]. However, to date, we do not have pathogen-resistant pepper plants in which a specific gene is edited.

Many plant-transformation studies have been performed with *Agrobacterium tumefaciens* as nature’s genetic engineer [11,12]. T-DNA binary vector systems in *A. tumefaciens* are the critical players for transforming plants. The T-DNA was engineered by deleting the oncogenes and the opine synthase genes to harness effective horizontal gene transfer from *Agrobacterium* to plants, effectively disarming the virulent strains so that tumors are not to induced [13,14]. Several non-oncogenic recombinant *Agrobacterium* strains currently popular in plant biotechnology include LBA4404, GV3101::pMP90, AGL1, EHA101, and its derivative strain, EHA105 [15]. As representative vegetables in Solanaceae, both tobacco and tomato have well-established *Agrobacterium*-mediated stable transformation systems, to conduct horticultural research and produce valuable cultivars for the horticultural industry [16,17,18,19,20,21,22]. However, *Capsicum*, another important genus of Solanaceae, is recalcitrant to transformation applied using advanced plant biotechnologies and still highly dependent on traditional breeding combined with molecular breeding [10,23].

Among the five domesticated species of *Capsicum—C. annuum*, *C. baccatum*, *C. chinense*, *C. frutescens*, and *C. pubescens* [24]—*C. annuum* is the most common and extensively cultivated pepper. These peppers are grouped into hot pepper and bell pepper that provide flavor, various pungency levels, and a variety of nutrients [24]. Although various transformation methods have been used in peppers, there are only a few successful cases, such as forming a callus-induced shoot with an inbred line, pepper cotyledon-derived or hypocotyl-derived in vitro regeneration methods, and developing *Phytophthora*-resistant transgenic *C. annuum* cv. Mesilla Cayenne plants [25,26,27,28]. Therefore, to improve pepper transformation, it is essential to apply the most appropriate breeding technologies [29,30,31,32]. Some of the factors affecting the efficiency of *Agrobacterium* strains in transforming tissues is the type of strain, for instance, octopine (LBA4404), succinamopine (AGL1 and EHA105), nopaline (EHA101, GV3101), and the vector [33,34,35,36,37], so it is crucial to validate which *Agrobacterium* strain is optimal for desired crops and their cultivars. For example, EHA101 and LBA4404 showed higher transformation efficiencies for local pepper genotypes Balady and Anaheim chile than GV3101 [38]. Although it is pivotal to validate the *Agrobacterium* strain harboring CRISPR tools so that successfully edited peppers can be obtained, there is still a lack of a sustainable pepper transformation system using CRISPR tools.

Here, we employed three strains of *A. tumefaciens*—AGL1, EHA101, and GV3101—to investigate which *Agrobacterium* strain could be used for pepper transformation. The whole-genome-sequenced hot pepper CM334 and bell pepper Dempsey were chosen in this study. Pepper cultivars are known to have different sensitivities to various selection markers [39]. Thus, we evaluated a suitable phosphinothricin (PPT) concentration to select the pBAtC binary vector harboring pepper transformants and analyzed *CaMLO2*, the target gene.

## 2. Results

### 2.1. Vector Construction and Agrobacterium-Mediated Transformation

**Figure 1:**
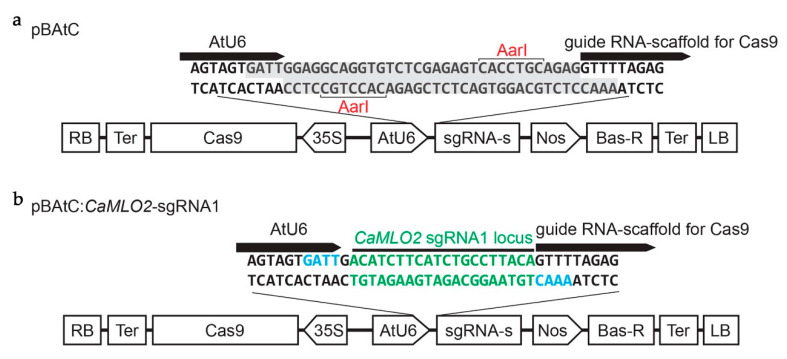
*CaMLO2* sgRNA1 cloned pBAtC binary vector. (**a**) Description of a pBAtC binary vector. RB, right border; Ter, tetracycline; CAS9hc:NLS: HA, human-codon-optimized Cas9 with the nuclear localization signal and an HA epitope; 35S, cauliflower mosaic virus (CaMV) 35S promoter; AtU6, *Arabidopsis thaliana* U6 promoter; Aar1, sgRNA cloning sites with two Aar1; sgRNA-s, the guide RNA-scaffold for Cas9; Nos, nos promoter; Bas-R, BASTA resistance gene; LB, left border; (**b**) Description of a pBAtC:*CaMLO2*–sgRNA1 binary vector. Green, the sgRNA1 sequence of *CaMLO2* target locus; Blue, overhangs digested by Aar1 sites.

We employed a pBAtC binary vector having a whole CRISPR/Cas9 cassette to edit a target gene in hot pepper and bell pepper [40]. Using a DNA-free, CRISPR/guide RNA screening system, we have previously selected an effective sgRNA1 (5′-ACATCTTCATCTGCCTTACA-3′) to target the *CaMLO2* gene in both cv. CM344 and Dempsey [10]. Using Aar1 sites, we constructed a pBAtC:*CaMLO2*–sgRNA1 vector and confirmed the guide RNA sequence using Sanger sequencing (Figure 1). To identify an effective *Agrobacterium* strain for hot pepper and bell pepper, the validated pBAtC:*CaMLO2*–sgRNA1 vector was transformed into each of three *A. tumefaciens* strains: AGL1, EHA101, and GV3101.

### 2.2. Comparison of Agrobacterium-Mediated Callus Induction in cv. Dempsey and CM334

**Figure 2:**
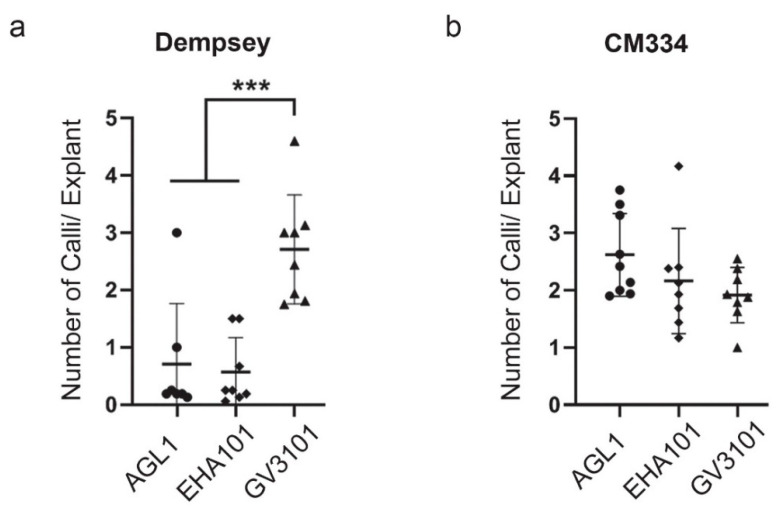
Comparison of callus induction ratios among the three *Agrobacterium* strains in *Agrobacterium*-mediated transformation of two pepper types. (**a**) Callus induction ratios of cv. Dempsey by three *Agrobacterium* strains (AGL1, *n* = 7 biological replicates (BR); EHA101, *n* = 8 BR; GV3101, *n* = 8 BR). Total number of explants was 265. Results are presented as mean ± SD; (**b**) Callus induction ratios of cv. CM334 by three *Agrobacterium* strains (AGL1, *n* = 9 BR; EHA101, *n* = 8 BR; GV3101, *n* = 8 BR). Total number of explants was 328. Results are presented as mean ± SD. Callus size ≥2.5 mm. ***, *p* < 0.001 based on analysis of variance (ANOVA).

We previously reported leaf-induced callus lines from hot pepper CM334 and bell pepper Dempsey for in vitro tissue culture and cell biological research [41]. Therefore, we adapted these pepper-leaf-induced calli for the current study. We took leaf explants from both cv. CM334 and Dempsey for *Agrobacterium*-mediated transformations using three different *Agrobacterium* strains (AGL1, EHA101, GV3101). We analyzed a total of 265 leaf explants of cv. Dempsey (86 with AGL1, 92 with EHA101, 87 with GV3101), and 328 leaf explants of cv. CM334 (119 with AGL1, 103 with EHA101, 106 with GV3101). The *Agrobacterium*-mediated callus induction numbers produced by all three *Agrobacterium* strains studied in the two types of pepper are summarized in Table S1. Numbers of induced calli (larger than 2.5 mm) of the transformants were measured for 4 weeks. Callus induction frequencies of six to eight biological replicates for each of the three *Agrobacterium* strains were analyzed. In cv. Dempsey, GV3101 generated an average of 2.7 calli per explant, more than EHA101 with 0.6 calli/explant and AGL1 with 0.7 calli/explant (Figure 2a). In cv. CM334, the average number of calli (2.6 with AGL1, 2.2 with EHA101, and 1.9 with GV3101) was similar among the three strains (Figure 2b). Therefore, GV3101 had the best callus-inducing activity for cv. Dempsey. For cv. CM334, all three *Agrobacterium* strains had comparable callus-inducing activities. These results suggest that the callus induction rate by an *Agrobacterium* strain can differ depending on the pepper cultivar.

### 2.3. Suitable Phosphinothricin (PPT) Concentration for Screening Transformants in cv. Dempsey and CM334

**Figure 3:**
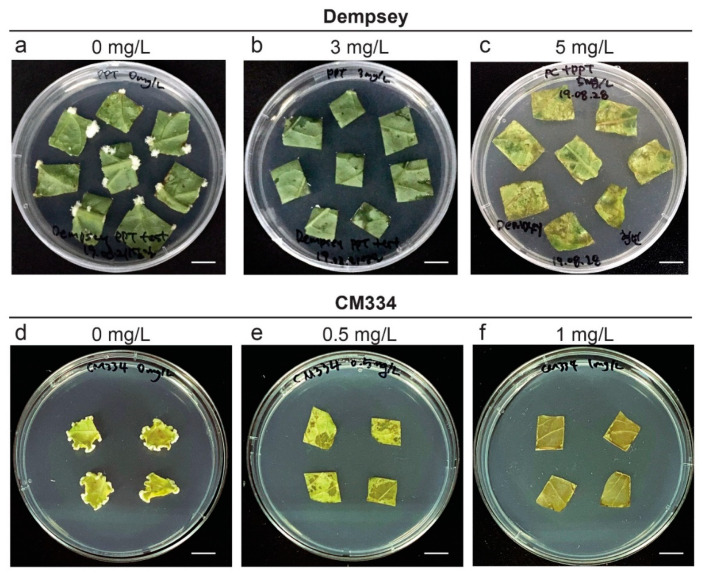
Effects of PPT on selection of callus of explants transformed by three different strains of *Agrobacterium*. (**a–c**) Examination of a suitable concentration of PPT for cv. Dempsey leaf explants. Cultivar Dempsey leaf explants were placed on CIM without PPT (0 mg/mL) (**a**), and with PPT at 3 mg/L (**b**) and 5 mg/L (**c**); (**d–f**) Examination of a suitable concentration of PPT for cv. CM334 leaf explants. Cultivar CM334 leaf explants on CIM without PPT (0 mg/mL) (**d**), and with PPT at 0.5 mg/L (**e**) and 1 mg/L (**f**) are shown. All explants on the indicated PPT medium were examined for 10 days. Scale bars = 1 cm. PPT, phosphinothricin; CIM, callus induction medium.

Peppers have different sensitivities to various selection markers, ranging from 0.05 mg/L methotrexate and 1 mg/L PPT to 25 mg/L hygromycin [39]. We examined the proper selection pressure by PPT on calli induced by pBAtC in transgenic hot pepper and bell pepper. We tested various concentrations of PPT (0.5, 1, 3, 5, 10 mg/L) for cv. Dempsey and CM334. In cv. Dempsey, the leaves treated with 3 mg/L PPT were not browned. They showed less callus induction than leaves treated without PPT. However, after treatment with 5 mg/L PPT, leaf explants were partially brown, and no callus had been induced (Figure 3a–c). By contrast, cv. CM334 leaves treated with 0.5 mg/L PPT were already partially browned, and callus rarely emerged (Figure 3e). In the presence of 1 mg/L PPT, cv. CM334 leaves were completely brown without induced callus (Figure 3f). These results showed that hot pepper CM334 was more sensitive to PPT than bell pepper Dempsey (Figure 3). If treated leaves were brown without further induction of an emerging callus and eventually died in the presence of PPT at a certain concentration, that concentration was considered the appropriate selection level. Therefore, we screened cv. Dempsey using 5 mg/L PPT and used 1 mg/L PPT to screen cv. CM334.

After performing *Agrobacterium*-mediated pBAtC:*CaMLO2*-sgRNA1 transformation in both pepper types using three different strains of *Agrobacterium* (AGL1, EHA101, and GV3101), we initially obtained different numbers of induced calli larger than 2.5 mm: 30, 37, 205 for cv. Dempsey; 325, 205, 205 for cv. CM334, respectively. These induced calli were selected for 30 days with bi-weekly subcultures on PPT media. It is noteworthy that the initially induced calli were selected in the PPT-containing media and differentially proliferated or decreased during the subculture processes. Therefore, we finally obtained 51, 68, and 63 calli in cv. Dempsey and 99, 52, and 65 in cv. CM334 for *Agrobacterium* strain AGL1, EHA101, and GV3101, respectively.

### 2.4. Evaluation of PPT-Selected Transformants in cv. Dempsey and CM334

**Figure 4:**
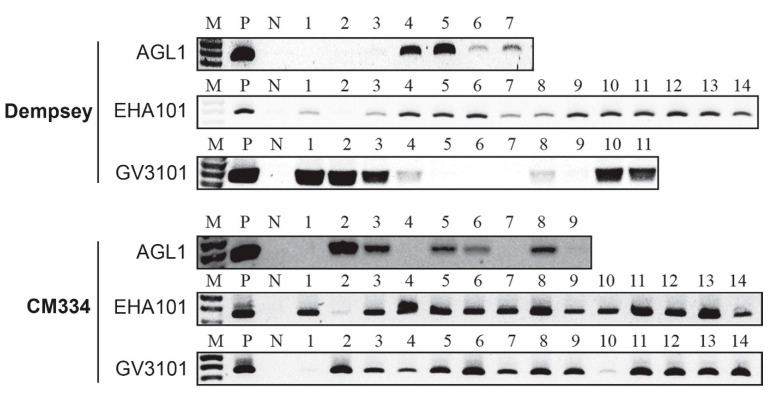
PCR analyses of PPT-selected and transformed calli of cv. Dempsey and CM334. (**a**) Calli in cv. Dempsey induced by *Agrobacterium* strain AGL1, EHA101, and GV3101, respectively; (**b**) calli in cv. CM334 induced by *Agrobacterium* strain AGL1, EHA101, and GV3101, respectively. M, 100-bp DNA ladder; P, pBAtC:*CaMLO2*–sgRNA1 binary vector; N, non-transformed pepper callus. The indicated numbers (1 to 14) are the PPT-selected and transformed calli in cv. Dempsey and CM334.

We also investigated whether these PPT-selected calli contained the pBAtC:*CaMLO2*–sgRNA1 binary vector. Each *Agrobacterium*-induced callus was analyzed by PCR with a specific primer pair—AtU6 promoter (forward) and guide RNA-scaffold (reverse)—targeting the pBAtC binary vector sequence (Figure 4). Thus, we validated PCR-positive pepper calli with the inserted sgRNA1 region of 362-bp in length (Figure 4). We applied PCR to a total of 102 cv. Dempsey calli and 107 cv. CM334 calli among the PPT-selected ones (Table 1). The percentage of PCR-positive transformants obtained from cv. Dempsey was 79.3% with AGL1, 61.1% with EHA101, and 51.4% with GV3101 and that from cv. CM334 was 75.7% with AGL1, 85.7% with EHA101, and 94.3% with GV3101 (Table 1). Thus, we were able to identify the true-positive calli of both pepper types by combining PPT selection and target-specific PCR analysis.

### 2.5. Analysis of CRISPR/Cas9 Indel Frequencies of Positive Transformants in cv. Dempsey and CM334

**Figure 5:**
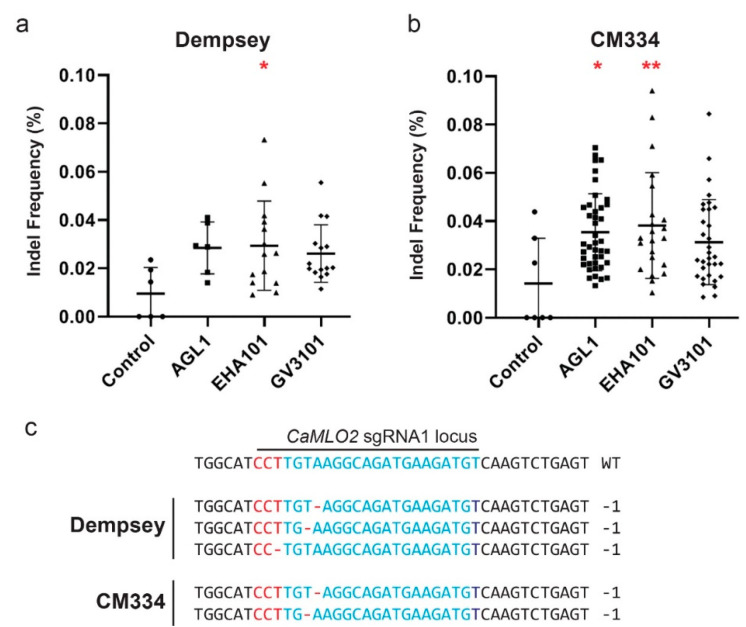
Comparison of indel frequencies of selected pepper calli following *Agrobacterium*-mediated transformation. (**a**) Indel frequencies of selected cv. Dempsey calli (Control, *n* = 6; AGL1, *n* = 6; EHA101, *n* = 14; GV3101, *n* = 15); (**b**) Indel frequencies of selected cv. CM334 calli (Control, *n* = 7; AGL1, *n* = 41; EHA101, *n* = 22; GV3101, *n* = 32). Callus size ≥2.5 mm. The indel frequency (%) was calculated by dividing the number of sequencing reads containing indel at the target site by the number of total sequencing reads. *, *p* < 0.05; **, *p* < 0.01 based on analysis of variance (ANOVA); (**c**) Indel patterns of selected calli in both cv. Dempsey and CM334. Red, PAM sequence; Blue, Cas9 target sequence; Red dashed lines (-), a deleted nucleotide.

We finally obtained both PPT- and PCR-positive transformed calli from 35 cv. Dempsey and 95 cv. CM334 using three different strains of *Agrobacterium* in *Agrobacterium*-mediated transformation. The numbers of double-positive transformants obtained for cv. Dempsey (Figure 5a, Table S2) and CM334 (Figure 5b, Table S2) were 6 and 41, 14 and 22, and 15 and 32 calli induced by *Agrobacterium* strain AGL1, EHA101, and GV3101, respectively. These selected calli were extracted for the preparation of genomic DNA (gDNA) from pepper and sequenced to investigate *CaMLO2* editing by targeted deep sequencing. Indel frequency (%) was calculated as the number of measured reads at the target locus divided by the number of total reads. The analyzed indel frequencies of the *CaMLO2* target locus from all double-selected transformants in both pepper types are summarized in Table S2. The indel frequency in cv. Dempsey was higher for calli transformed by *Agrobacterium* strain EHA101 than strains AGL1 or GV3101, with *p* = 0.0184 compared to control non-transformants (Table S3). However, the differences among all three strains were marginal, with an average frequency of 0.028% and the highest frequency of 0.07% (Figure 5a, Table S2). Cultivar CM334 showed similar indel frequencies to cv. Dempsey, with an average frequency of 0.035% and the highest frequency of 0.09% with EHA101 (Figure 5b, Table S2). The EHA101 strain was slightly better than the AGL1 and GV3101 strains for target gene editing in cv. CM334 (Figure 5b). The performed statistical analyses are described in Table S3.

Although the indel frequencies in the target *CaMLO2* gene of transformed calli were not as high as those shown in protoplast-based systems at more than 10%, the editing at the target locus occurred very precisely with 1-bp deletion at the sgRNA1 locus of *CaMLO2* (Figure 5c). Moreover, indel patterns at the target locus were reproducible throughout the 130 selected double-positive transformants of cv. Dempsey and CM334 (Figure 5c). These results demonstrate that generating a gene-edited pepper cultivar is challenging but feasible with optimized transformation strategies and CRISPR tools.

## 3. Discussion

*Agrobacterium tumefaciens* is a soilborne, pathogenic, Gram-negative bacterium that causes plants to produce crown gall disease following the transfer, integration, and expression of oncogenes by the T-DNA region of the tumor-inducing (Ti) plasmid [42]. The compatibilities between pepper and *Agrobacterium* strains are dependent on the pepper cultivar [43]. We found that *Agrobacterium* GV3101 resulted in a higher callus induction rate than AGL1 and EHA101 in pepper cv. Dempsey, whereas all three *Agrobacterium* strains showed similar rates of callus induction in pepper cv. CM334. These results confirmed that both hot pepper Dempsey and bell pepper CM334 also had different compatibilities with *Agrobacterium* strains. The nopaline-type *A. tumefaciens* GV3101 is recommended for *Arabidopsis thaliana* floral-dip and root-transformation methods [44] and could thus be assumed to be the best strain for cv. Dempsey too. However, when the pBAtC:*CaMLO2*–sgRNA1 binary vector harboring *Agrobacterium* strains was used to infect cv. Dempsey, the most effective strain for inducing calli in cv. Dempsey was not GV3101. This result was validated by both PPT selection and PCR analysis. Although the GV3101 strain effectively induced calli in cv. Dempsey, these calli were not truly transformed, but false-positively proliferated. Therefore, the effective callus-induced strain did not correlate with the strain having the best editing frequency.

The most desired outcome for pepper editing is to have 100% edited without any chimera patterns of the target gene. We could not detect such a high indel frequency among the three *Agrobacterium* strains tested in this study. Only marginal efficiency was found. However, we determined that *Agrobacterium* EHA101 was the best among the tested strains for cv. Dempsey and CM334 to obtain statistically significant editing efficiency at the target locus *CaMLO2* when using at least seven biological replicates. Although several disarmed *Agrobacterium* strains, such as LBA4404 or GV3101, are frequently used in generating genetically modified crops, improvement of current *Agrobacterium* strains or discovery of new “super-virulent” strains will be essential for recalcitrant crops, such as hot pepper and bell pepper [42,45].

Considering that the indel frequency in the editing of both pepper types can be more than 10% with active complexes of Cas9 protein and sgRNA1 in protoplasts [10], the pepper genome itself is not problematic for applying CRISPR tools. We confirmed that the delivered pBAtC binary vector was detected by PCR analysis in PPT-selected calli. However, the binary vector may not be efficient enough to express Cas9 and sgRNA1, the two key players of the CRISPR system in pepper cv. CM334 and Dempsey. Therefore, a binary vector can be improved by a pepper-favorable CRISPR-expressing cassette to produce more active components in peppers.

The functional relevance of *CaMLO2* in the disease resistance of peppers has already been investigated [46]. Although the genome-editing era is well underway, *CaMLO2*-edited peppers are not yet available due to the recalcitrance of pepper. Therefore, it is essential to have a confirmed CRISPR delivery strategy to generate genome-edited inheritable peppers. Here, we demonstrate that *A. tumefaciens* strain EHA101 is the most optimal one to transform hot pepper CM334 and bell pepper Dempsey among the three *A. tumefaciens* tested (AGL1, EHA101, and GV3101). Based on the confirmed efficacy of sgRNA1 for the *CaMLO2* gene, we successfully delivered the CRISPR/Cas9 binary vector system called pBAtC:*CaMLO2*–sgRNA1 into cv. CM334 and Dempsey. We provide proper concentrations of PPT as a selective marker of pBAtC at 1 mg/L for cv. CM334 and 5 mg/L for cv. Dempsey.

## 4. Materials and Methods

### 4.1. Plant Materials

Hot pepper, *C. annuum* cv. CM334 (Criollo de Morelos 334), a landrace collected from the Mexican state of Morelos [7], and bell pepper, *C. annuum* cv. Dempsey, a cultivar originating from a three-way cross between PI163192, PI264281, and Jupiter cultivars [47], were provided by the Vegetable Breeding Research Center (VBRC) in Seoul, the Republic of Korea. Pepper leaf explants were used from 6-week-old *C. annuum* L. cv. Dempsey and CM334 for *Agrobacterium*-mediated transformation. Pepper seeds were sterilized with 2% commercial bleach and 0.1% Tween-20 for 20 min and washed three times with distilled water for 10 min each time. These surface-sterilized seeds were germinated on Murashige-Skoog (MS) medium with vitamins (Duchefa Biochemie, Haarlem, Netherlands), 2% sucrose, and 0.8% phytoagar with pH adjusted to 5.8. These sowed plates were incubated at 25 °C for 1 week in the dark. Germinated pepper seedlings were grown at 25 °C with 60% humidity under 16 h light and 8 h dark photoperiods in a growth chamber (HANKUK S&I, Korea) for 5 weeks.

### 4.2. Plasmid Construction

The target locus of the *CaMLO2* gene from both pepper types, sgRNA1 (5´-ACATCTTCATCTGCCTTACA-3′), was screened using a DNA-free CRISPR/guide RNA screening system [10]. The selected sgRNA1 was cloned into a pBAtC vector via Aar1 sites [40]. The cloned sgRNA1 sequence was confirmed by Sanger sequencing (Macrogen, Seoul, Korea).

### 4.3. Agrobacterium-Mediated Transformation

Cloned binary pBAtC:*CaMLO2*–sgRNA1 vector was transformed into each strain of *A. tumefaciens* AGL1, EHA101, and GV3101. These three strains were incubated in 4 mL YEB, containing 50 mg/L spectinomycin and 25 mg/L rifampicin for 48 h to obtain seed culture. As the main culture, 100 mL YEB (50 mg/L spectinomycin, 25 mg/L rifampicin) was inoculated with 2 mL of each seed culture and incubated at 28 °C with shaking at 180 rpm overnight to obtain an optical density at 600 nm (OD_600_) of 1.0. After overnight growth, these three strains were diluted to OD_600_ of 0.3 using harvest buffer (2.2 g/L MS medium including vitamins, 0.9 mg/L thiamin, 39 mg/L acetosyringone, 1% sucrose, pH 5.8) for co-cultivation. Leaf explants (1.5 × 1.5 cm) were placed in the *Agrobacterium* suspension and co-cultured at 25 °C and 60% humidity for 30 min. Leaf explants were removed, wiped thoroughly with 3M paper, and then cultured on 3M paper wetted with the harvest buffer for 48 h at 25 °C and 60% humidity in the dark.

### 4.4. Callus Induction

Co-cultured explants were placed on a callus induction medium (CIM) (for cv. Dempsey: 2.4 g/L MS medium basal salt mixture including MES buffer, 0.4 mg/L thiamin, 0.1 g/L *myo*-inositol, 3% sucrose, 0.8% phytoagar, 500 mg/L cefotaxime, pH 5.8; for cv. CM334: 3.1 g/L Gamborg’s B5 medium including vitamins, 0.5 g/L MES monohydrate, 2.0 mg/L 6-benzylaminopurine, 1.0 mg/L of 1-naphthaleneacetic acid, 3% sucrose, 0.8% phytoagar, 500 mg/L cefotaxime, pH 5.8 [41]. The number of induced calli (larger than 2.5 mm) among the transformants was measured for 4 weeks and analyzed for callus induction.

### 4.5. Antibiotic Selection

PPT (Duchefa Biochemie, Haarlem, Netherlands) was dissolved in deionized water to 10 mg/mL as a stock and sterilized by filtration through a 0.2 μm filter (Satorius, Sungnam, Korea). The indicated concentration of PPT in the PPT-included CIM for either cv. Dempsey or CM334 was freshly prepared before the co-cultivating leaf explants. Co-cultivated leaves were placed on the PPT-included CIM for 10 days. Negative transformants were observed to have browned leaves with rare emerging calli that eventually died in the presence of PPT for 10 days. Positive transformants were detected if there was proliferative callus at the diced edge of leaf explants.

### 4.6. Genomic DNA (gDNA) Extraction from Pepper and PCR Analysis

gDNA of both pepper types was extracted from selected calli by the CTAB method [48]. To validate the existence of the pBAtC:*CaMLO2*–sgRNA1 vector in selected calli, we used a specific pair of primers—forward (5′-GAATGATTAGGCATCGAACC-3′) and reverse (5′-AAAAAAGCACCGACTCGG-3′)—to amplify the inserted sgRNA1 region with a length of 362 bp.

### 4.7. Targeted Deep Sequencing

The indel frequency and patterns of pepper transformants were analyzed by targeted deep sequencing [10]. The gDNA was extracted from pepper transformants and non-transformants of both pepper types and amplified with specific primers (Table 2) to read the target *CaMLO2* locus in cv. CM334 and Dempsey. The gDNA was used to construct the target amplicon libraries by consecutive PCRs to add multiplexing indices and sequencing adaptors. The amplicon library was sequenced using the Illumina MiSeq V2 Reagent Kit (300-cycle; San Diego, CA, USA) to monitor indels at the target locus. Raw data of paired-end MiSeq were analyzed by running Cas-Analyzer (http://www.rgenome.net/cas-analyzer/#! accessed on 5 April 2021), a CRISPR RGEN tool for assessing genome editing results using next-generation sequencing data [49]. The indel frequency (%) was calculated by dividing the number of sequencing reads containing indel at the target site by the number of total sequencing reads. The indel patterns at the target *CaMLO2* locus were retrieved from the sequenced raw data of transformants from both pepper types analyzed.

### 4.8. Statistical Analysis

Presented data were statistically analyzed using GraphPad Prism 8.0. (San Diego, CA, USA) The significance of the data was investigated through a *t*-test or analysis of variance (ANOVA) (*, *p* < 0.05; **, *p* < 0.01, ***, *p* < 0.001).

## Figures and Tables

**Table 1:** Summary of the percentages of positive (PCR and PPT) transformants in cv. Dempsey and CM334.

	Cultivar	Dempsey			CM334		
*Agrobacterium*		Number of PCR-Applied Calli among PPT Selected	Number of PCR-Positive Calli	Percentage of both Positives (PPT and PCR)	Number of PCR-Applied Calli among PPT Selected	Number of PCR-Positive Calli	Percentage of both Positives (PPT and PCR)
AGL1		29	23	79.3	37	28	75.7
EHA101		36	22	61.1	35	30	85.7
GV3101		37	19	51.4	35	33	94.3

**Table 2:** Primers used in targeted deep sequencing.

Primer	Sequence
*CaMLO2* F	ATGGCTAAAGAACGGTCGAT
*CaMLO2* R	ATGGAGCTGGTGTATTGCAT
Primary F	TGGGATTCATATCATTGTTGTTG
Primary R	CCGAATGTGTCTCAGCCTTT
Secondary F	ACACTCTTTCCCTACACGACGCTCTTCCGATCTTGGGATTCATATCATTGTTGTTG
Secondary R	ACTGGAGTTCAGAGTGTGCTCTTCCGATCTCCGAATGTGTCTCAGCCTTT

F; forward; R, reverse.

## Data Availability

All data supporting reported results can be found in the article.

## Supplementary Materials

The following are available online at https://www.mdpi.com/article/10.3390/ijms22083921/s1, Table S1: The summary of three *Agrobacterium* strains-mediated callus induction in Dempsey and CM334, Table S2: The summary of indel frequencies of *CaMLO2* sgRNA1 locus from all transformants of Dempsey and CM334, Table S3: The summary of statistical analyses of *CaMLO2* edited transformants in Dempsey and CM334 peppers.
