# Supplementary material for: Agrobacterium-Mediated Capsicum annuum Gene Editing in Two Cultivars, Hot Pepper CM334 and Bell Pepper Dempsey
Source: Int J Mol Sci. 2021 Apr 10;22(8):3921. doi: 10.3390/ijms22083921 (PMC8070316; doi:10.3390/ijms22083921)
Supplement: Supplementary file 1 [file ijms-22-03921-s001.pdf]

Agrobacterium-Mediated *Capsicum annuum* Gene Editing in Two Cultivars, Hot Pepper CM334 and Bell Pepper Dempsey

Sung-il Park [1], Hyun-Bin Kim [2], Hyun-Ji Jeon [2] and Hyeran Kim [1,2,*]

Supplementary Data

Table S1. The summary of three *Agrobacterium* strains-mediated callus induction in Dempsey and CM334.

Cultivar / *Agrobacteria*	Dempsey			CM334		
	Explants	Numbers of induced callus	Numbers of callus / Explant	Explants	Numbers of induced callus	Numbers of callus / Explant
AGL1	16	4	0.25	10	20	2
	16	3	0.19	16	56	3.5
	16	3	0.19	12	29	2.42
	16	3	0.19	7	15	2.14
	15	2	0.13	16	53	3.31
	3	3	1	16	60	3.75
	4	12	3	10	19	1.9
	.	.	.	16	42	2.63
	.	.	.	16	31	1.94
EHA101	16	3	0.19	15	36	2.4
	8	2	0.25	15	32	2.13
	16	4	0.25	12	14	1.17
	6	9	1.5	6	25	4.17
	6	4	0.67	8	19	2.38
	8	12	1.5	15	29	1.93
	16	2	0.13	16	27	1.69
	16	1	0.06	16	23	1.44
GV3101	16	31	1.94	14	27	1.93
	16	39	2.44	14	25	1.79
	5	23	4.6	8	15	1.88
	8	25	3.13	16	35	2.19
	16	28	1.75	11	28	2.55
	16	29	1.81	11	11	1
	4	12	3	16	38	2.38
	6	18	3	16	26	1.63

Table S2. The summary of indel frequencies of *CaMLO2* sgRNA1 locus from all transformants of Dempsey and CM334. Gray box, indicated the mean of above column each.

Cultivar / Strain / Sample	Indel frequency of Dempsey (%)				Indel frequency of CM334 (%)			
	Control	AGL1	EHA101	GV3101	Control	AGL1	EHA101	GV3101
1	0.01935609	0.018386	0.041872	0.020033	0.02271695	0.050714	0.018193	0.017197
2	0.02354419	0.029448	0.073292	0.011471	0	0.065378	0.032923	0.029206
3	0	0.038865	0.055325	0.019446	0	0.028149	0.033038	0.022199
4	0	0.014039	0.017425	0.016474	0	0.04669	0.035818	0.017512
5	0.01436885	0.041079	0.025846	0.028432	0.0438943	0.020385	0.027349	0.044984
6	0	0.028891	0.026302	0.02907	0	0.067367	0.020065	0.057192
7	**0.00954485**	**0.02845133**	0.018746	0.04182	0.03293808	0.049056	0.082919	0.045746
8			0.009019	0.055516	**0.01422133**	0.025332	0.037005	0.025199
9			0.013908	0.017596		0.070442	0.021971	0.017043
10			0.014045	0.030297		0.013263	0.059198	0.022415
11			0.009979	0.020296		0.01599	0.054635	0.024584
12			0.036284	0.021988		0.040945	0.071004	0.026969
13			0.030245	0.018322		0.057212	0.025031	0.022211
14			0.039274	0.019867		0.016441	0.030919	0.012785
15			**0.02939729**	0.041517		0.027297	0.017463	0.008566
16				**0.026143**		0.020884	0.094009	0.01517
17						0.044	0.040527	0.00907
18						0.022694	0.03882	0.023914
19						0.027547	0.031955	0.020587
20						0.027742	0.010339	0.023199
21						0.022372	0.042741	0.044853
22						0.04154	0.01526	0.050753
23						0.016369	**0.03823555**	0.04707
24						0.031698		0.038146
25						0.022812		0.065989
26						0.031071		0.047676
27						0.046893		0.034462
28						0.065232		0.039692
29						0.034204		0.013857
30						0.030544		0.032773
31						0.024656		0.016106
32						0.029303		0.084495
33						0.022043		**0.03130063**
34						0.025183		
35						0.019986		
36						0.060775		
37						0.045741		
38						0.039185		
39						0.017119		
40						0.042333		
41						0.04551		
						**0.035417**		

Table S3. The summary of statistical analyses of *CaMLO2* edited transformants in Dempsey and CM334 peppers.

a

Ordinary one-way ANOVA of Dempsey indel

Number of families	1
Number of comparisons per family	3
Alpha	0.05

Dunnett's multiple comparisons test	Mean Diff.	95.00% CI of diff.	Significant?	Summary	Adjusted P Value	A-?	
Control vs. AGL1	-0.01891	-0.03888 to 0.001069	No	ns	0.0664	B	AGL1
Control vs. EHA101	-0.01985	-0.03673 to -0.002970	Yes	*	0.0184	C	EHA101
Control vs. GV3101	-0.0166	-0.03331 to 0.0001146	No	ns	0.0519	D	GV3101

Test details	Mean 1	Mean 2	Mean Diff.	SE of diff.	n1	n2	q	DF
Control vs. AGL1	0.009545	0.02845	-0.01891	0.008278	6	6	2.284	37
Control vs. EHA101	0.009545	0.0294	-0.01985	0.006996	6	14	2.838	37
Control vs. GV3101	0.009545	0.02614	-0.0166	0.006926	6	15	2.397	37

b

Ordinary one-way ANOVA of CM334 indel

Number of families	1
Number of comparisons per family	3
Alpha	0.05

Dunnett's multiple comparisons test	Mean Diff.	95.00% CI of diff.	Significant?	Summary	Adjusted P Value	A-?	
Control vs. AGL1	-0.0212	-0.03805 to -0.004338	Yes	*	0.0111	B	AGL1
Control vs. EHA101	-0.02401	-0.04190 to -0.006126	Yes	**	0.0064	C	EHA101
Control vs. GV3101	-0.01708	-0.03428 to 0.0001206	No	ns	0.0519	D	GV3101

Test details	Mean 1	Mean 2	Mean Diff.	SE of diff.	n1	n2	q	DF
Control vs. AGL1	0.01422	0.03542	-0.0212	0.007392	7	41	2.867	98
Control vs. EHA101	0.01422	0.03824	-0.02401	0.007843	7	22	3.062	98
Control vs. GV3101	0.01422	0.0313	-0.01708	0.007542	7	32	2.265	98
